# Exposing additional authors who suppress evidence about radiation-induced thyroid cancer in children: a Comment adding to Tsuda et al.’s response to Schüz et al. (2023)

**DOI:** 10.1186/s12940-023-01033-3

**Published:** 2023-11-16

**Authors:** Colin L. Soskolne

**Affiliations:** School of Public Health, University of Alberta, Edmonton, AB Canada

**Keywords:** Bias, Epidemiologic methods, Evidence-based public health, Fukushima, Nuclear fallout, Professional ethics, Retraction recommended

## Abstract

**Background:**

The need to call out and expose authors for their persistence in improperly using epidemiology has been previously noted. Tsuda et al. have done well to expose Schüz et al.’s arguments/assertions in their recent publication in *Environmental Heath*. In this Comment, I point out that, also warranting being called out, are the arguments/assertions of Cléro et al. who, in their recent response to an article by Tsuda et al., reiterated the conclusions and recommendations derived from their European project, which were published in *Environment International* in 2021.

Tsuda et al. had critiqued the Cléro et al. 2021 publication in their 2022 review article. However, in their response to it, Cléro et al. deflected by not addressing any of the key points that Tsuda et al. had made in their review regarding the aftermath of the Chernobyl and Fukushima nuclear accidents. In this Comment, I critique Cléro et al.’s inadequate response.

Publication of this Comment will help in routing out the improper use of epidemiology in the formulation of public health policy and thereby reduce the influence of misinformation on both science and public policy. My critique of Cléro et al. is not dissimilar from Tsuda et al.’s critique of Schüz et al.: in as much as Schüz et al. should withdraw their work, so should Cléro et al.’s article be retracted.

**Main body:**

The response by Cléro et al. consists of four paragraphs. First was their assertion that the purpose of the SHAMISEN project was to make recommendations based on scientific evidence and that it was not a systematic review of all related articles. I point out that the Cléro et al. recommendations were not based on objective scientific evidence, but on biased studies.

In the second paragraph, Cléro et al. reaffirmed the SHAMISEN Consortium report, which claimed that the overdiagnosis observed in non-exposed adults was applicable to children because children are mirrors of adults. However, the authors of that report withheld statements about secondary examinations in Fukushima that provided evidence against overdiagnosis.

In the third paragraph, Cléro et al. provided an explanation regarding their disclosure of conflicting interests, which was contrary to professional norms for transparency and thus was unacceptable.

Finally, their insistence that the Tsuda et al. study was an ecological study susceptible to “the ecological fallacy” indicated their lack of epidemiological knowledge about ecological studies. Ironically, many of the papers cited by Cléro et al. regarding overdiagnosis were, in fact, ecological studies.

**Conclusion:**

Cléro et al. and the SHAMISEN Consortium should withdraw their recommendation “not to launch a mass thyroid cancer screening after a nuclear accident, but rather to make it available (with appropriate information counselling) to those who request it.” Their recommendation is based on biased evidence and would cause confusion regarding public health measures following a nuclear accident. Those authors should, in my assessment, acquaint themselves with modern epidemiology and evidence-based public health. Like Tsuda et al. recommended of Schüz et al., Cléro et al. ought also to retract their article.

## Background regarding Cléro et al.’s response to Tsuda et al.

From the SHAMISEN (Nuclear Emergency Situations—Improvement of Medical and Health Surveillance) international experts’ consortium, Cléro et al. published a review article entitled, “Lessons learned from Chernobyl and Fukushima on thyroid cancer screening and recommendations in the case of a future nuclear accident” [1]. Their review presented principles of cancer screening, lessons learned from thyroid cancer screening, findings regarding thyroid cancer incidence after Iodine-131 exposure, and it provided recommendations “not to launch a mass thyroid cancer screening after a nuclear accident, but rather to make it available (with appropriate information counselling) to those who request it”.

Their review, however, misrepresented the Fukushima empirical findings and the Chernobyl experience by including citations of biased literature and by emphasizing overdiagnosis in thyroid screening using ultrasound echo, but with no specific verification/validation. Tsuda et al. communicated the current situation in Fukushima and Japan, pointed out errors in Cléro et al.'s claims, and evaluated those claims using the “Toolkit for detecting misused epidemiologic methods” [2, 3]. Cléro et al. responded to Tsuda et al.’s 2022 review paper [3] and provided their own comments [4]. I read with concern Cléro et al.’s response [4] regarding Tsuda et al.’s previous review article [3]. In this Comment, I provide my assessment of their review [4] and the SHAMISEN Consortium [1].

## Comment on the response by Cléro et al. [4]

Cléro et al. responded that the SHAMISEN paper was not a systematic review of all papers published on the Chernobyl and Fukushima accidents [1, 4]. However, Tsuda et al. had pointed out that the Cléro et al. review was biased in its selectiveness of studies to support their position [3]. Some well-known papers were not cited, even though these were introduced in Tsuda et al.’s previous review paper [3].

Cléro et al. stressed that the evidence suggests that overdiagnosis is similar in children and adults [4]. However, what is mirrored with adults, as described by Vaccarella et al. [5], is only the incidence and epidemiological pattern of thyroid cancer in adults and children/adolescents, not overdiagnosis itself. The definition of overdiagnosis is not standardized among authors [1, 5–8]. As noted in Tsuda et al.’s comments [9] on Schüz et al. [10], there is no evidence to demonstrate overdiagnosis of thyroid cancer in children, in general. Vaccarella et al.'s method shows differences between the expected and observed rates, and they concluded that a larger area under the observed curve indicated greater overdiagnosis in adults [8]. For children and adolescents [5], as indicated above, Vaccarella et al. changed the definition of overdiagnosis to the definition used for adults; thus, their claims regarding overdiagnosis are not backed by relevant evidence.

Cléro et al. emphasized "small thyroid nodules" in cases of overdiagnosis [1], but, because individuals with small nodules were excluded from the ultrasound examinations in Fukushima, the definition of overdiagnosis in Fukushima is not related to nodule size [3]. The use of ultrasound echo limited the number of individuals to those with a nodule of 5.1 mm or larger, which was found among 2,293 test positives out of 300,472 (i.e., 1 in 131) examinees during the first round of screening (2011–2014) [11]; these individuals then underwent secondary examinations, including active surveillance extending for more than several months. In the secondary examination, the number of individuals with a nodule of 5.1 mm or larger reduced to 1 in 3.9 [11]; these people then underwent Fine Needle Aspiration Cytology (FNAC), if necessary. Among examinees with abnormal findings, the proportion who underwent FNAC was 4.3% (257/6,009 up to June 30, 2022, in a total of four rounds of screening between 2011–2020) [11]. Therefore, overdiagnosis of thyroid cancer was even less likely to occur by ultrasound echo. Cléro et al. did not evaluate this process at all in their critique [1, 4]. Concealment of this fact would give readers the false impression of overdiagnosis.

Cléro et al. described the way that Dr. Dominique Laurier declared her conflict-of-interest [1, 3, 4]. They did not contradict the statement by Tsuda et al. regarding Cléro et al.'s failure to disclose conflicting interests, only that they did not fail to do so, as stated in *Environment International* [1, 3, 4]. Cléro et al. did not explain why they did not cite published articles that provide important information on this issue; nor did they address why they distorted the Fukushima information. I hope that *Environment International,* and the public at large, will carefully assess whether the claims of Cléro et al. are valid. Furthermore, conflicting interests should be disclosed that involve IARC Expert Group chairs and the Japanese government, whose evident bias is to obscure the large excess incidence of thyroid cancer in Fukushima.

## Study design, causal inference, and the role of epidemiologists

In the final paragraph before their conclusion, Cléro et al. [4], using Jorgensen’s letter against the Tsuda et al. article [12], insisted that Tsuda et al.’s study design was an ecological study and, as such, was susceptible to “the ecological fallacy.” As explained by Tsuda et al., Fukushima Medical University (FMU) designed that study as a cohort study [3, 13]. Cléro et al. did not comment regarding how the results and conclusions were specifically affected by the study design, nor did they mention how many of the papers cited in their review on overdiagnosis in fact used an ecological study design [1]. Those researchers might do better to consider the Tsuda et al. study a cohort study; however, others might disagree. According to the classification by Morgenstern [14], the study design of the Tsuda et al. paper was a semi-ecological analysis. Their rationale explains why ecological studies are widely used in epidemiological studies. Two examples follow as questions:

- First, if the epidemiological study on historical air pollution in six cities in the United States by Dockery et al. were considered to have a semi-ecological rather than a cohort study design [15], then could it be argued that the US Environmental Protection Agency should not have made changes to air pollution standards in the United States based on results of that six-city study [16]?
- Second, if the design of Snow's foundational epidemiological investigation regarding the outbreak of cholera [17] were deemed a semi-ecological study, could it be claimed that the Southwark and Vauxhall Waterworks Company should not have relocated its Thames River intake?

The main role of epidemiologists is not to classify study designs; nor is the role of epidemiologists to continue to insist on things for which there is no evidence. The role of epidemiologists is to collect and analyze data, draw conclusions based on the results of analyses that take the study design employed into account, and make valid causal inferences based on the body of evidence. Then, the role of epidemiologists in public health is to propose and implement necessary and feasible intervention measures in a timely manner, based on these results and conclusions. Cléro et al. [1, 4], the SHAMISEN Consortium [1], Jorgensen [12], and even Schüz et al. [6, 10] appear to me to not understand the development of epidemiology during the past half century and the importance of public health measures. Unfortunately, they have failed to revise their position considering changes in the field [18].

## Conclusion

In their conclusion, Cléro et al. claimed, without evidence, that the large number of excess thyroid cancers in Fukushima was caused by overdiagnosis [1, 4]. Although their argument is centered on overdiagnosis, those authors were unable to disprove that the severe Fukushima Daiichi Nuclear Power Plant accident that occurred in March 2011 was the cause of excess thyroid cancers, in accordance with the existing evidence [3, 5, 9]. Consequently, they are hindering the implementation of public health measures in Fukushima Prefecture and worsening the health outcomes of patients, especially those with latent thyroid cancer.

Without sufficient discussion and evidence on the already disproven claim regarding overdiagnosis and the effects of screening, the above authors and the SHAMISEN Consortium have caused distress and confusion and have delayed scientifically justifiable interventions in Japan [1, 6, 9]. These authors should understand that overdiagnosis has not occurred in children [1, 5, 6, 8, 9]. Additionally, and most importantly, these authors ought, in my view, to become better acquainted with ethical conduct in modern epidemiology and evidence-based public health. Given that the thyroid gland is exquisitely sensitive to the radioactive isotope, for example, Iodine-131, to argue that thyroid cancer excesses can be explained predominantly by overdiagnosis exhibits a clear bias, unbecoming of public health professionals.

Finally, members of the SHAMISEN Consortium appear to me to have a deep understanding of the Japanese language in which the phrase "playing shamisen" is Japanese slang; it means "to mislead" or "to lie” [19]. Again, such conduct would be unbecoming of public health professionals. Cléro et al.’s article ought to be retracted.

## Data Availability

All the data necessary to reproduce the results reported in this Comment are available on the Fukushima Prefecture website (Reference 11).

## Abbreviations

IARC: International Agency for Research on Cancer
SHAMISEN: Nuclear Emergency Situations - Improvement of Medical and Health Surveillance
FNAC: Fine Needle Aspiration Cytology
UNSCEAR: United Nations Scientific Committee on the Effects of Atomic Radiation
